# Comparison of Bone Mineral Density between Urban and Rural Areas: Systematic Review and Meta-Analysis

**DOI:** 10.1371/journal.pone.0132239

**Published:** 2015-07-10

**Authors:** Mika Matsuzaki, Rashmi Pant, Bharati Kulkarni, Sanjay Kinra

**Affiliations:** 1 Department of Non-communicable Disease Epidemiology, London School of Hygiene and Tropical Medicine, London, United Kingdom; 2 Indian Institute of Public Health, Hyderabad, India; 3 National Institute of Nutrition, Hyderabad, India; Garvan Institute of Medical Research, AUSTRALIA

## Abstract

**Background:**

Studies from high income countries (HIC) have generally shown higher osteoporotic fracture rates in urban areas than rural areas. Low bone mineral density (BMD) increases susceptibility to fractures. This review aimed to assess whether urbanicity is consistently associated with lower BMD globally.

**Method:**

Ovid MEDLINE, EMBASE, and Global Health (-April 2013) were searched for articles investigating differences in bone mineral content (BMC) or BMD between urban and rural areas. Ratio of means (RoM) of BMD were used to estimate effect sizes in meta-analysis, with an exception for one study that only presented BMC data.

**Results:**

Fifteen articles from eleven distinct populations were included in the review; seven populations from four high income countries and four from three low and middle income countries (LMIC). Meta-analysis showed conflicting evidence for urban-rural difference in BMD; studies from high income countries generally showed higher BMD in rural areas while the results were more mixed in studies from low and middle income countries (HIC RoM = 0.05; 95% CI: 0.03 to 0.06; LMIC RoM = -0.04: 95% CI: -0.1 to 0.01).

**Conclusions:**

Urban-rural differences of bone mineral density may be context-specific. BMD may be higher in urban areas in some lower income countries. More studies with robust designs and analytical techniques are needed to understand mechanisms underlying the effects of urbanization on bone mass accrual and loss.

## Introduction

Morbidity and mortality associated with hip fracture is a major public health concern [1–4]. Suboptimal bone mineral density (BMD), muscle weakness, impaired balance and cognition can all contribute to osteoporotic hip fracture [4]. Bone mass accrual and loss are influenced by a number of modifiable risk factors throughout life, including dietary intake of calcium and protein, serum vitamin D level, and weight-bearing physical activity [5,6].

A previous systematic review showed moderate evidence for lower fracture rates in rural areas compared to urban areas [7]. Most of the studies in this review were from high income countries (HIC), as defined by the World Bank [8], due to better availability of reliable fracture records. However, the prevalence of osteoporotic fracture has been rising in low and middle income countries (LMIC), where rapid urbanization has also been taking place [9]. Lifestyles, especially dietary patterns and physical activity levels, generally vary between urban and rural areas but how they differ may be context-specific, especially in relation to stages of economic development at country level [10]. There is therefore a need to examine the effect of urbanicity on bone mass accrual and loss globally.

Bone densimetry tools like dual-energy x-ray absorptiometry (DXA) have been used for the assessment of bone mass and density in both HICs and LMICs. These data enable assessment of association between urbanicity and BMD in a global context. Bone mass data are also available from wider age groups than osteoporotic fracture records, allowing assessment of the effect of urbanicity in younger populations as well.

We assessed the evidence on comparison of bone mineral density between urban and rural areas in meta-analyses and examined any variation in patterns of urban-rural differences among countries at differing stages of economic development.

## Method

The Preferred Reporting Items for Systematic Reviews and Meta-Analysis checklist and flow diagram were referred to structure this manuscript [11].

### Search Strategy

We reviewed articles investigating differences in bone mineral density or content for adults (≥ 20 years) as well as children and adolescents (<20 years) living in urban or rural areas. Search terms used for bone outcomes were “bone mass”, “bone mineral density”, “bone mineral content”, BMD, and BMC. Terms “osteoporosis” and “osteopenia” were also included in the initial search in order to be more inclusive although they were not primary outcomes of interest. In addition, we searched for studies including terms for “urban” or “rural”.

Appropriate wild cards were used to account for use and non-use of space and dashes. We searched the MEDLINE, EMBASE, and GLOBAL HEALTH electronic databases for full articles published before April 2013. We excluded the following publication types: Historical Article or News or Newspaper Article or Review, Multicase or Review, Tutorial or Review of Reported Cases (OVID Medline), Review (OVID EMBASE), and Patent (OVID Global Health). The complete search strategies are listed in the Supporting Information S1 Table.

### Study selection and inclusion criteria

**Fig 1:**
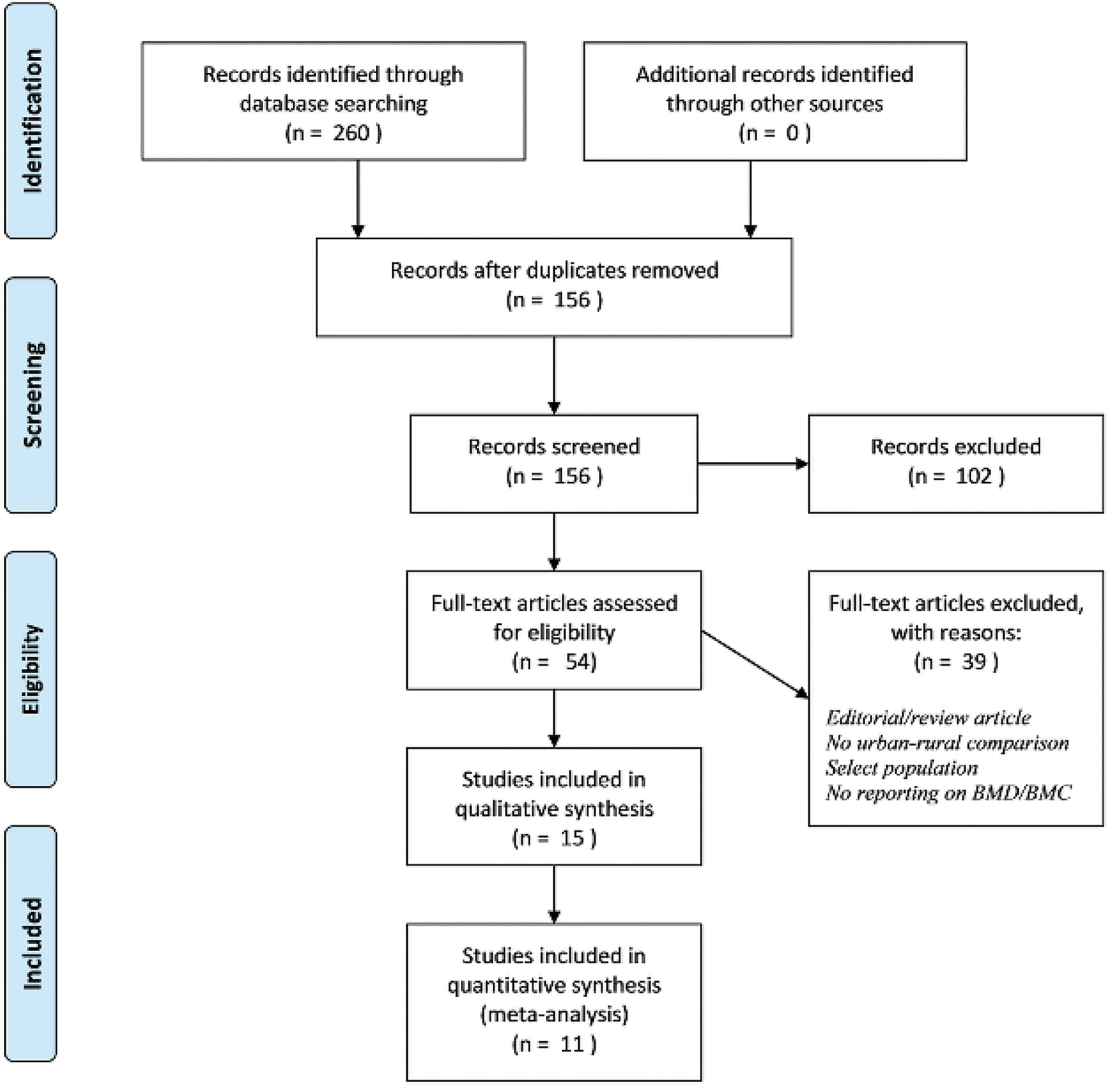
Flow diagram of study selection process from initial search to included studies.

We included studies that compared bone mineral density or content between urban and rural areas in the same country (**Fig 1**). Duplicate articles were removed (n = 104) and then two reviewers independently examined the titles and abstracts for inclusion. Articles with only abstracts available or articles written in non-English languages were excluded. Full articles were examined when it was not clear from the titles or abstracts whether comparison of BMC or BMD in urban and rural areas was done. The discrepancies (n = 14) were resolved in a consensus meeting. Inter-rater agreement was assessed using kappa coefficients (κ) [12].

### Data extraction

Data extraction was done by two independent reviewers. We extracted the following data: country, year of the study, definitions of urban or rural areas, sample size (n), age, mean and standard deviation (sd) of BMD (or BMC if BMD data were not available) in urban and rural area, and, where available, results of statistical comparison between urban and rural groups. The economic status of the countries at the time of study were identified, using the World Bank’s classification system, and categorized into either low and middle income countries or high income countries [8,13]

The Thai Epidemiological Study had three articles based on the same study population and four independent sets of data were extracted, which differed in sex (men and women) and sites of bone mass measurements (hip and lumbar spine) [14,15]. For men in the Thai Epidemiological Study, BMD data from the same study population were presented in two articles. We used the data from the 2006 article for the meta-analyses as the article had a slightly higher sample size for the rural population (duplicated data for the urban population).

### Data analysis

When data from sub-groups within each sex, such as age groups and pre/postmenopausal women, were presented in articles, the data were pooled into one group to conduct statistical comparison between urban and rural areas. All hip data were based on femoral neck measurements. The analyses in this review were done separately for high income countries and low and middle income countries.

We used the ratio of means (RoM) between rural and urban populations to allow comparison of results from studies using different instruments [16,17]. RoM of BMD was calculated for all but one study, which only presented BMC data [18]. The natural logarithm of RoM and its SE were calculated for each study for the analysis. Random effects models controlling for heterogeneity in between-study variation were tested. The heterogeneity of effects across studies were estimated by Q test. Publication bias was assessed by a funnel plot and Egger's test. Q test, funnel plot, and Egger's test were only performed for HIC papers as there were too few studies from LMIC. All statistical analyses were performed in R version 3.1.1.

## Results

### Search results and study types

The initial search yielded 260 articles, of which 54 articles were found potentially eligible for inclusion after title and abstract search. In the full text search, 39 articles were further excluded because of one of the following reasons: they only had BMC or BMD data for either urban or rural areas; only osteoporosis prevalence data were available; only abstracts were available; or the articles were written in non-English language. A total of 15 articles met our selection criteria (**Fig 1**) [14,15,18–30]. Inter-rater agreement was high; two reviewers (MM and RP) scored 156 items and agreed on 137 (87%, κ = 0.62). No other articles were identified through hand-searching of the reference lists of these 15 articles. There were three articles published using the same population from the Thai Epidemiological Study [14,15,27]. These three articles provided four datasets (hip and lumbar spine bone mass measurements for men and for women) for our meta-analyses. There were two articles based on a study population in Malmö, Sweden, and two articles from the Norwegian Epidemiological Osteoporosis Studies (NOREPOS) [14,15,19,24–27]. A study by Gärdsell *et al* included only BMC data [18]. One HIC study did not provide sample sizes for urban and rural populations [29].

All studies had difficulty blinding researchers from urban and rural locations as the bone mass measurements were typically done within the towns where participants resided. The only cohort study included in this review was a large-scale, multi-decade study from Sweden and had low participation rates [19,24]. **Table 1** shows the characteristics of the included studies. Three studies were conducted within the last decade [14,15,22,23,27]. Six studies were from low and middle income countries (China [23,28], Thailand [14,15,27], and Sri Lanka [22]) and nine studies were from high income countries (Norway [25,26], Sweden [19,21,24,30], Poland [20], and the United States of America (USA) [29]). The age range of the study participants was from 11 to 89 years. There were two studies that examined adolescents under 20 years old [21,22]. All studies analyzed BMC and/or BMD in urban and rural areas for each sex separately. The majority of the articles were based on dual x-ray absorptiometry (DXA) data (n = 8) while five articles used single photon absorptiometry (SPA) and two articles used single x-ray absorptiometry (SXA). Bone mass measurements in all studies were done on the same type of bone densitometer within each study. There were nine studies whose main research question included urban and rural comparison of BMC/BMD [14,18,20–23,25,29].

**Table 1:** Study characteristics of the included articles.

National Income Level	Country; Study name	First author; Study year	Age range; sex	Sample size	Bone mass measurement device	Urban and rural definitions
**LMIC**	Thailand;	Pongchaiyakul [15];	20–84;	n = 872	DXA	U: Bangkok, a capital city, a population of 5.7 million, lifestyle similar to that in Western cities.
	Thai Epidemiological Study	2005	men and women			R: Khon Kaen, a province, a population of 1.8 million, considered one of the most typical agricultural communities in Thailand.
		Pongchaiyakul [27];	20–87; men	n = 412	DXA	(same as above)
		2005				
		Pongchaiyakul [14];	20–84;	n = 847	DXA	(same as above)
		2006	men and women			
	China;	Wanli [28];	>60;	n = 470	SPA	No definition given. All from Hongmen country of Xinxiang city.
		2005	men and women			
	China;	Gu [23];	50–70;	n = 1179	DXA	U: a city with an official urban residential (non-agricultural) registration
		2007	men and women			R: a village of a county with an agricultural residential registration according to the Chinese residential registration system
	Sri Lanka;	Ranathunga [22];	11–16;	n = 1181	DXA	U: Colombo
		2008	girls			R: Pannala
**HIC**	Norway;	Omsland [26];	>65;	n = 7333	SXA	Based on the population density of the election district (refers to Meyer *et al*):
	NOREPOS	2011	women			U: urban Tromsø;
						R: rural Tromsø (additionally, the rural region included Nord-Trøndelag, a rural county with a few small villages.)
		Meyer [25];	40–75;	n = 10,667	SXA	Based on the population density of the election district:
		2004	men and women			U: urban Tromsø
						R: rural Tromsø
	Sweden;	Sundberg [21];	15–16;	n = 250	DXA	U: a suburb of the city of Malmo, population size of 245,000, population density of 1595 inhabitants/km^2^, the third largest city in Sweden.
		1997	boys and girls			R: Hassleholm County, population size of 50,000, 38 inhabit/km^2^
	Sweden;	Ringsberg [30];	65–89;	n = 165	SPA	U: the city of Malmo, the third largest city in Sweden, population size of 240,000, a centre of trade and industry.
		2001	women			R: Sjobo, a typical agricultural community
	Sweden;	Rosengren [19];	50–80;	(1988/89)n = 437	SPA	Based on the national population records:
		2010	women	(1998/99) n = 289		U: the city of Malmo, population size of 230,383 in 1987 and 265,481 in 2002.
						R: nine rural municipalities near the country village Sjobo, all predominantly agricultural municipalities, population size of 134,458 in 1987 and 141,989 in 2001.
		Rosengren [24];	50–80;	(1988/89) n = 323	SPA	(same as above)
		2012	men	(1998/99) n = 141		
	Sweden;	Gardsell [18];	≥40;	n = 961	SPA	Based on the Central Bureau of Statistics:
		1991	men and women			U: Malmo, the third largest city in Sweden, population size of 231,575 in 1988, a typical Swedish urban population.
						R: Sjobo, population size of 15,350 in 1988, considered one of the most typical agricultural communities in Sweden.
	Poland;	Filip [20];	30–79;	n = 503	DXA	U: Lublin urban area
		2001	women			R: Urzędów district, 40km from the nearest town, lack of industry, significant percentage of farmers.
	USA;	Specker [29];	20–66;	n = 1189	DXA	Based on the Rural-Urban Continuum Codes for South Dakota used by the U.S. Census Bureau:
	South Dakota Rural Bone Health Study	2004	men and women			Non-rural: population size of 2500 to 19,999.
						R: completely rural or population size of less than 2500.
						Hutterite: isolated communal living, agricultural-based rural lifestyle.

LMIC: low and middle income countries; HIC: high income countries; SPA: single photon absorptiometry; SXA: single-energy x-ray absorptiometry; DXA: dual-energy x-ray absorptiometry; U: Urban; R: Rural

There was a wide variation in the definition of urban and rural areas as shown in Table 1: one study gave no definition [28]; one study only gave the names of the urban and rural areas [22]; one study used rural features such as 40km from the nearest town, lack of industry, and high farming practice [20]; one study was based on the national residential registration for agricultural and non-agricultural areas [23]; all other studies used census data of population size or density, with some additionally describing the patterns in agricultural practice. One study compared physically active urban population to rural population as well as non-active urban population [30]. Another study compared two rural sub-populations, Hutterite population, who is an isolated religious community that engages in self-sufficient lifestyle through agriculture, and non-Hutterite rural population [29].

**Fig 2:**
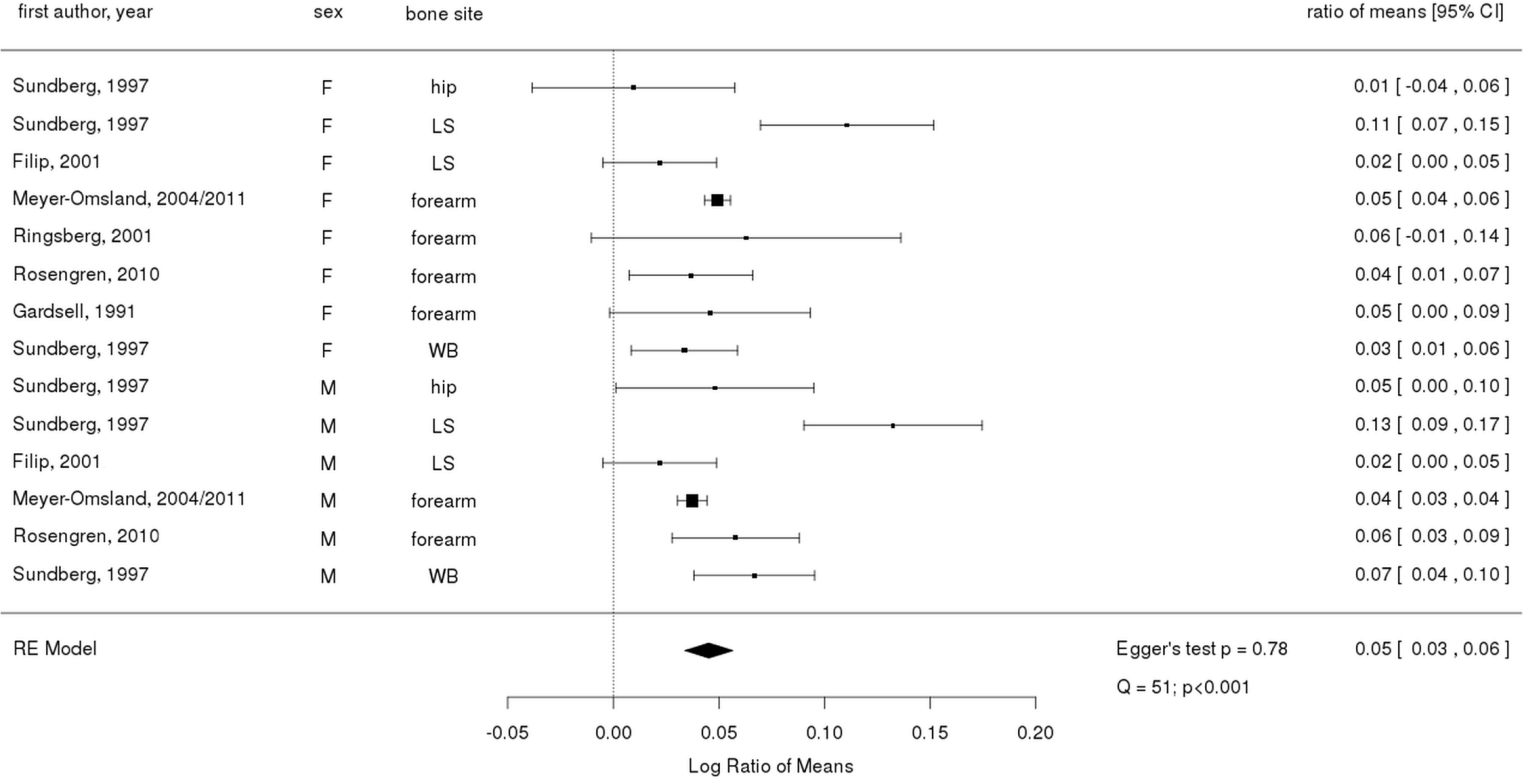
Ratio of means and 95% confidence interval for comparing bone mineral content or density in urban and rural populations in high income countries. Symbol sizes are proportional to sample sizes. The overall effect size was derived from a random-effects model. LS: lumbar spine. WB: whole body. F: female. M: male.

**Fig 3:**
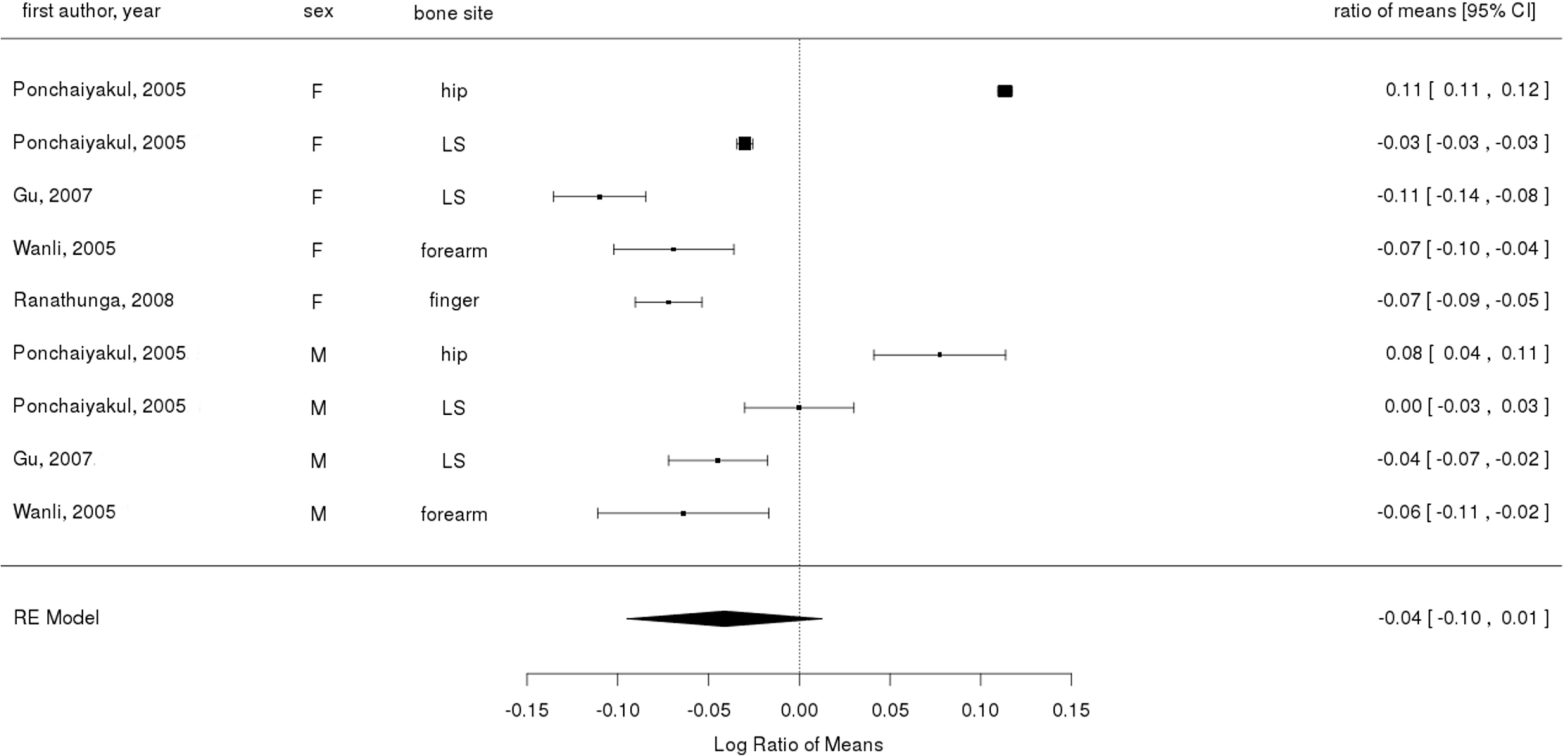
Ratio of means and 95% confidence interval for comparing bone mineral content or density in urban and rural populations in low and middle income countries. Symbol sizes are proportional to sample sizes. The overall effect size was derived from a random-effects model. LS: lumbar spine. WB: whole body. F: female. M: male.

**Fig 2** (HIC) and **Fig 3** (LMIC) show the meta-analysis of BMD in urban and rural populations, with the exception of BMC comparison by Gärdsell *et al* [18]. There were five articles examining hip, seven articles lumbar spine, five articles forearm, one article finger, and one article total body. Since both 1988/89 and 1998/99 data in Rosengren’s cohort study showed similar patterns in BMD differences between urban and rural areas, the values from two time points were pooled for meta- analysis.

The pooled analysis showed that rural residents had a 5% higher BMD than urban residents in HIC (RoM = 0.05; 95% CI: 0.03 to 0.06). On the other hand, studies from LMICs showed mixed results (RoM = -0.04: 95% CI: -0.1 to 0.01). Publication bias for HIC studies did not indicate any systematic trend of publication bias (Egger's test p = 0.78). There was between-study heterogeneity found for HIC studies (Q = 51; p <0.001). Publication bias and heterogeneity were not tested for LMIC studies as there were too few studies.

## Discussion

Three out of four studies from low and middle income countries provided evidence that bone mineral density in urban areas is higher than rural areas while there was no study from high income countries that showed higher BMD in urban areas.

### Comparison with previous research

Our findings from HIC studies are generally in line with the moderate evidence found in a previous systematic review for lower risk of osteoporotic hip fracture among rural residents [7]. Mixed results in LMIC found here are in concordance with a view expressed previously on osteoporosis and urbanicity in LMIC [31]. The discrepancy in findings between HIC and LMIC shown in this review suggests that the effect of urbanization may be context-specific. There is a possibility that further economic development in LMIC may shift patterns of association between urbanicity and BMD more towards negative associations seen in HIC through environmental and individual lifestyle changes.

There are a number of potential lifestyle and environmental factors contributing to healthier bone development in rural areas in HIC, including higher physical activity level, higher serum vitamin D level, and less air pollution. A handful of studies conducted further statistical analyses to show how these risk factors may be associated with regional differences in BMD. In high income countries, longer sedentary time, lower micronutrient intake, and lower BMI are generally considered to be characteristics of urban dwellers. In Norway, urban women had lower BMI than rural women and body size adjustment attenuated the BMD differences between urban and rural areas in women [25]. Physical activity level or smoking status did not explain the regional differences. In Sweden, urban women who have engaged in regular exercise activity for twenty years had higher BMD in comparison to urban women who did not regularly exercise [30]. The difference between rural women and active urban women was less clear. In the South Dakota Rural Bone Health Study (SDRBHS), all rural residents engaged in more than 75% of their life on a farm and spent less than 1040 hours a year on non-farming work. Although there were differences in BMD between rural and non-rural populations, current physical activity level, dietary intake of calcium and vitamin D, or muscle strength did not explain these population differences. The authors speculated that the higher physical activity level during childhood and adolescence in the farming rural population may be partially responsible for the observed difference.

On the other hand, in lower income countries, urban residents may have better bone health profiles as they have better access to food, education, jobs, and social welfare that may not be available in rural areas. The studies from China showed higher BMC and BMD in urban areas [23]. Gu *et al* showed that the urban and rural difference was attenuated in men upon adjustment for body size, suggesting that higher body mass due to better nutrition may contribute to higher BMD in urban area [23]. However, for women, the urban and rural difference persisted even after adjusting for body size, income, milk consumption, calcium and vitamin D supplement intake, total physical activity, walking, and social activity. The Thai Epidemiological Study explored whether regional differences in lean and fat mass explained BMD differences [15]. Rural residents had higher lean mass and lower fat mass but did not always show higher BMD when compared to urban residents. The matched pair analysis in men showed that lean mass explained more of the variance of urban and rural difference in BMD than fat mass. Although lean mass was positively associated with BMD in women as well, the urban and rural difference in lean mass did not account for the differences in BMD as much as in men.

### Strengths and limitations

There are some limitations to this review. Only full articles were reviewed while there were several conference abstracts describing urban-rural differences in BMD. Articles written in non-English languages were also excluded, which is likely to have reduced the number of articles from LMIC included in this study. For instance, Gu *et al* discusses four papers written in Chinese that showed a range of findings for urban-rural differences in BMD or the prevalence of osteoporosis in China.

The number of studies included in this review was fairly small. More studies, especially from LMIC, are needed in order to ascertain our observation on differences in urban and rural areas between HIC and LMIC. Because there were only seven countries included in this review, the interaction between national income levels and urba-rural differences could not be tested statistically formally and therefore, the conclusion should be treated with caution. Similarly to Brennan’s review, the definitions of urban and rural area varied considerably among studies, which also urges careful interpretation of the results. Ten out of eleven studies were based on cross-sectional data limiting causal inference between urbanicity and bone mineral density. More cohort studies are needed in order to determine how urban and rural lifestyles and environments may influence bone mass accrual and loss throughout life.

There were also very few studies examining children and adolescents. While there were more studies examining younger adults (<50 years), most studies focused on the elderly. If lifestyles during the bone development phase are indeed important as suggested in the SDRBHS, there needs to be more studies on how lifestyle and environmental changes due to urbanization may be associated with bone development in younger populations. Suboptimal bone mineral density is a major contributing factor for osteoporotic hip fracture. Since body size is strongly associated with bone mineral density, better food availability in cities may be beneficial for bone development in lower income countries, at least during the initial phase of economic transition. Low physical activity level and excess food intake are more commonly observed among urban dwellers in higher income countries in comparison to urban areas in lower income countries. As the epidemic of osteoporosis continues to grow globally, effects of urbanization on bone health in LMICs and HICs ought to be carefully examined in order to develop appropriate interventions.

#### Summary Box

What is already known on this subject?

- Bone mineral density is a key determinant of osteoporotic fractures.
- Whether BMD is higher in urban than rural areas globally is not known

What does this study add?

- Bone mineral density was higher in urban areas in some low and middle income countries while no high income countries showed higher BMD in urban areas.
- There may be different underlying mechanisms of the effects of urbanization on bone mineral density in countries at various economic stages.

## Supporting Information

S1 Checklist PRISMA statement. (DOC)

S1 Dataset Sample size and means of bone mineral content/density for meta-analyses. (CSV)

S1 Table Summary of search strategy. (DOC)
