# Correction: Comparison of Bone Mineral Density between Urban and Rural Areas: Systematic Review and Meta-Analysis

**DOI:** 10.1371/journal.pone.0137151

**Published:** 2015-08-26

**Authors:** Mika Matsuzaki, Rashmi Pant, Bharati Kulkarni, Sanjay Kinra

The following information is missing from the Funding section. The publication of this review was supported by the Wellcome Trust (EPNCBD6710). The funders had no role in study design, data collection and analysis, decision to publish, or preparation of the manuscript.
