# Supplementary material for: Comparison of Bone Mineral Density between Urban and Rural Areas: Systematic Review and Meta-Analysis
Source: PLoS One. 2015 Jul 10;10(7):e0132239. doi: 10.1371/journal.pone.0132239 (PMC4498744; doi:10.1371/journal.pone.0132239)
Supplement: S1 Table — (DOC) [file pone.0132239.s003.doc]

**Supporting Information Table S1: Summary of search strategy**

| Medline | EMBASE | Global Health |
| --- | --- | --- |
| 1. exp Bone Density/ or exp "Bone and Bones"/ or exp "Osteoporosis"/  2. (osteoporosis or osteopenia or bone mass or bone mineral density or bone mineral content or BMD or BMC).mp. [mp=title, abstract, original title, name of substance word, subject heading word, keyword heading word, protocol supplementary concept, rare disease supplementary concept, unique identifier]  3. 1 or 2  4. exp Urban Health/ or exp Urban Population/  5. exp Urbanization/  6. (urban$ or city or non-rural or nonrural).mp. [mp=title, abstract, original title, name of substance word, subject heading word, keyword heading word, protocol supplementary concept, rare disease supplementary concept, unique identifier]  7. 4 or 5 or 6  8. exp Rural Health/ or exp Rural Population/  9. rural$.mp. [mp=title, abstract, original title, name of substance word, subject heading word, keyword heading word, protocol supplementary concept, rare disease supplementary concept, unique identifier]  10. 8 or 9  11. (urban-rural or rural-urban).mp. [mp=title, abstract, original title, name of substance word, subject heading word, keyword heading word, protocol supplementary concept, rare disease supplementary concept, unique identifier]  12. bone.mp. [mp=title, abstract, original title, name of substance word, subject heading word, keyword heading word, protocol supplementary concept, rare disease supplementary concept, unique identifier]  13. limit 12 to abstracts  14. 7 and 10  15. 11 or 14  16. 3 and 13 and 15  17. limit 16 to (english language and humans)  18. 7 or 10 or 11  19. 3 and 13 and 18  20. limit 19 to (english language and humans)  21. (Historical Article or News or Newspaper Article or Review or Review, Multicase or Review, Tutorial or Review of Reported Cases).pt.  22. (17 not (Historical Article or News or Newspaper Article or Review or Review, Multicase or Review, Tutorial or Review of Reported Cases)).pt. | 1. exp bone density/  2. exp bone/ or exp bone development/ or exp osteoporosis/  3. (osteoporosis or osteopenia or bone mass or bone mineral density or bone mineral content or BMD or BMC or bone density).mp. [mp=title, abstract, subject headings, heading word, drug trade name, original title, device manufacturer, drug manufacturer, device trade name, keyword]  4. exp urban rural difference/ or exp urban population/  5. exp urbanization/  6. (urban$ or city or non-rural or nonrural).mp. [mp=title, abstract, subject headings, heading word, drug trade name, original title, device manufacturer, drug manufacturer, device trade name, keyword]  7. 4 or 5 or 6  8. exp rural population/  9. rural$.mp. [mp=title, abstract, subject headings, heading word, drug trade name, original title, device manufacturer, drug manufacturer, device trade name, keyword]  10. 8 or 9  11. (urban-rural or rural-urban).mp. [mp=title, abstract, subject headings, heading word, drug trade name, original title, device manufacturer, drug manufacturer, device trade name, keyword]  12. 7 and 10  13. 11 or 12  14. 1 or 2 or 3  15. 13 and 14  16. bone.mp. [mp=title, abstract, subject headings, heading word, drug trade name, original title, device manufacturer, drug manufacturer, device trade name, keyword]  17. limit 16 to abstracts  18. 15 and 17  19. limit 18 to (human and english language)  20. 7 or 10 or 11  21. 14 and 17 and 20  22. limit 21 to (human and english language)  23. Review.pt.  24. 22 not 23  25. 19 not 23 | 1. exp bones/ or exp osteoporosis/  2. exp bone density/  3. (osteoporosis or osteopenia or bone mass or bone mineral density or bone mineral content or BMD or BMC).mp. [mp=abstract, title, original title, broad terms, heading words]  4. 1 or 2 or 3  5. exp rural urban relations/ or exp rural urban migration/ or exp urban population/  6. urbanization.sh.  7. (urban$ or city or non?rural).mp. [mp=abstract, title, original title, broad terms, heading words]  8. 5 or 6 or 7  9. exp rural health/ or exp urban rural migration/ or exp rural depopulation/ or rural communities/ or exp rural population/  10. rural$.mp. [mp=abstract, title, original title, broad terms, heading words]  11. 9 or 10  12. 8 and 11  13. (rural-urban or urban-rural).mp. [mp=abstract, title, original title, broad terms, heading words]  14. 12 or 13  15. bone.mp. [mp=abstract, title, original title, broad terms, heading words]  16. limit 15 to abstracts  17. 4 and 14 and 16  18. limit 17 to english language  19. 8 or 11 or 13  20. 4 and 16 and 19  21. limit 20 to english language |
